# TLR2 was overexpressed independent of IL-6 in patients with valvular atrial fibrillation^☆^

**DOI:** 10.1016/S1674-8301(11)60023-7

**Published:** 2011-05

**Authors:** Jian Wang, Lei Xue, Hailong Cao, Fei Cui, Ting Dai, Yijiang Chen

**Affiliations:** a Department of Thoracic and Cardiovascular Surgery, the First Affiliated Hospital of Nanjing Medical University, Nanjing, Jiangsu 210029, China;; b Department of Pharmacology, Nanjing Medical University, Nanjing, Jiangsu 210029, China.

**Keywords:** Toll-like receptor 2, atrial fibrillation, inflammation, valvular heart disease

## Abstract

Toll-like receptor 2 (TLR2) has recently been shown to be up-regulated in patients with non-valvular atrial fibrillation (AF). The present study was aimed to determine whether the pathogenesis and development of AF is associated with the up-regulation of TLR2. Clinical data and right atrial appendage (RAA) specimens were collected from 20 patients with persisten AF (PeAF), 15 patients with paroxysmal AF (PaAF) and 13 patients with no history of AF undergoing valvular replacement. The results showed that gene expression and protein content of TLR2 were increased in both the AF subgroups, compared with the sinus rhythm (SR) group. Between the two AF subgroups, PaAF had a higher TLR2 level than PeAF. However, no difference in interluekin (IL)-6 content was found among the three groups, and no correlation was found between TLR2 and IL-6 in PeAF patients (*r* = 0.090, *P* = 0.706), PaAF patients (*r* = 0.408, *P* = 0.131) and AF patients (*r* = -0.301, *P* = 0.079). Immunohistochemical analysis revealed that TLR2 was distributed in RAAs of AF patients and confirmed the immunoblotting results. In conclusion, we demonstrated that TLR2 was elevated in AF (especially PaAF) patients with valvular heart disease, further implicating inflammation involved in the pathogenesis and development of AF.

## INTRODUCTION

Atrial fibrillation (AF), the most common arrhythmia seen in clinical practice[1], is associated with substantial morbidity and mortality. AF is generally categorized into valvular and non-valvular AF, depending on the presence of valvular heart disease. Various disorders, such as ischemic, valvular, inflammatory and degenerative heart diseases, as well as hormonal disorders and systemic hypertension, predispose to AF[2]–[6]. Furthermore, additional risk markers such as age, obesity, anger and hostility, and long-term alcohol consumption partly account for the increase in AF[7]–[13]. Although a number of risk factors have been associated with AF, acute or chronic hemodynamic, metabolic, and especially inflammatory stressors may lead to structural remodeling of the atria that may promote progression and persistence of AF. Moreover, inflammation seems to be intimately linked to the initiation and perpetuation of AF[14],[15]. One of the first studies comparing inflammatory markers to atrial remodeling was carried out by Frustaci *et al*.[16]. Furthermore, evidence for inflammation contributing to at least some types of AF was suggested by the frequent occurrence (25% to 40%) of AF after cardiac surgery[17]. Serum markers have been measured to assess the involvement of systemic inflammation in the initiation and promotion of AF and the development of cardioembolic stroke.

Recent studies have shown that Toll-like receptor (TLR) family members, which induce distinct patterns of gene expression, are key regulators of both innate immunity and antigen-specific acquired immunity[18]. Furthermore, although the roles of TLRs in human diseases are still not fully understood, there are significant results to support roles for particular TLRs in disease initiation and progression. Moreover, a loss-of-function study suggested that both TLRs contribute to myocardial inflammation and ischemic injury and modulate cell survival and tissue injury in the heart[19]. However, among 10 TLRs identified in humans, at least two exist in the heart, i.e., TLR2 and TLR4. TLR2, one isoform of TLRs, has also been studied in a multitude of models of human inflammatory diseases, such as arthritis and ulcerative colitis[20],[21]. Recently, TLR2 has been evaluated to be up-regulated in patients with non-valvular AF, which suggests that inflammation may participate in the pathogenesis of non-valvular AF. In the present study, we attempted to test our hypothesis that atrial levels of TLR2 were up-regulated to different degrees in paroxysmal AF (PaAF) and persistent AF (PeAF) patients with valvular heart disease.

## MATERIALS AND METHODS

### Patient population

Right atrial appendages (RAAs) were obtained from 48 patients undergoing isolated valve replacement in the First Affiliated Hospital of Nanjing Medical University. They were divided into three groups, 20 patients with PeAF, 15 patients with PaAF, and 13 patients with sinus rhythm (SR). Patients were excluded if they had 1) other organic disorders responsible for AF, such as diabetes mellitus, hyperthyroidism, or hypertension; 2) autoimmune diseases such as rheumatoid arthritis; 3) thromboembolic disease such as atherosclerosis; 4) systemic inflammatory diseases including infection, collagen diseases, malignancies, renal failure, and hepatic failure. ***Table 1*** describes detailed clinical characteristics of these subjects. Written informed consent was obtained from all patients, which was in accordance with a protocol approved by the ethics committee of Nanjing Medical University.

**Table 1:** Patient characteristics

Variables	SR (*n* = 13)	AF (*n* = 35)		*P*
		PeAF (*n* = 20)	PaAF (*n* = 15)	
Gender (male/female)	9/4	15/5	8/7	0.394
Age (yrs)	47.2±9.7	47.6±9.6	47.0±11.5	0.984
Heart rate (beats/min)	82±11	80±17	84±16	0.851
C-reactive protein (mg/L)	2.85±1.56	5.82±2.21**	5.10±2.71*	0.002
Duration of valvular disease (years)	3.8±4.5	8.7±6.9*	6.2±6.8	0.104
NYHAclass( I/II/III/IV)	0/7/4/2	0/8/7/5	3/3/7/2	0.123
Echocardiographic parameters				
LAD (mm)	44.3±8.6	60.4±8.1**^††^	52.1±8.6**	<0.001
LVDd (mm)	55.0±8.1	53.5±8.6	53.2±7.1**	0.815
LVDs (mm)	34.8±5.6	37.5±6.2	36.5±7.0**	0.495
EF (%)	62.7±4.7	61.4±5.7	61.5±7.9**	0.826
Preoperative length of stay (days)	15.3±7.8	21.8±8.5*	18.1±7.9**	0.086
Preoperative antiarrhythmics				
ACE inhibitor,n (%)	3(23)	6(30)	9(60)	0.087
Digitalis,n (%)	8(62)	17(85)	12(80)	0.278
Beta-blocker,n (%)	0	8**	4*	0.034
Ca^2+^-blocker,n (%)	0	4	2	0.235
Operative data				
Valve (MVR/ DVR/ AVR)	13(5/2/6)	20(10/10/0**)	15(10/4/1)	0.002

Values are presented as mean±SD or number of patients. *P < 0.05, ** *P* < 0.01 compared with the SR group; ^†^ *P* < 0.05, ^††^ *P* < 0.01 compared with the PaAF group. ACE-I: angiotensin-converting enzyme inhibitor; MVR: mitral valve replacement; AVR: aortic valve replacement; DVR: double valve replacement; EF: ejection fraction; LAD: left atrial diameter; LVDd: left ventricular end-diastolic diameter; LVDs: left ventricular end-systolic diameter; NYHA: New York Heart Association; PaAF: paroxysmal atrial fibrillation; PeAF: persistent atrial fibrillation; SR: sinus rhythm.

### Quantitative real-time PCR (qRT-PCR)

The primers for TLR2 and human glyceraldehyde-3-phosphate dehydrogenase (*GAPDH*) gene were synthesized by Invitrogen Co., Hong Kong, China. The sequences of primers used were as follows: for TLR2, forward 5′-ATCCTCCAATCAGGCTTCTCT-3′ and reverse 5′-ACACCTCTGTAGGTCACTGTTG-3′, and for GAPDH, forward 5′-ATGGGGAAGGTGAAGGTCG-3′ and reverse 5′-GGGGTCATTGA TGGCAACAATA-3′. Total RNA was extracted from the tissue of RAAs by the acid-phenol extraction method using the TRIzol reagent (Invitrogen, USA), and subsequently precipitated with isopropanol ethanol. Total RNA was used for synthesis of cDNA according to the manufacturer's instructions with the reverse transcriptase kit (Promega Co., USA). Human *TLR2* mRNA levels were determined by quantitative real-time PCR (qRT-PCR) using cDNA obtained from the reverse transcription reactions as template, which was performed using the ABI Prism 7000 sequence detection system (Applied Biosystems, CA, USA) with a total volume of 25 µL containing 12.5 µL 2× SYBR Premix Ex *Taq*™, 0.5 µL forward primers, 0.5 µL reverse primers, 0.5 µL 50× Rox Reference Dye, 1 µL cDNA template, and 10 µL H_2_O. Human *GAPDH* mRNA served as a control for the amount of cDNA present in each sample. The protocol consisted of 40 cycles, and the cycling parameters were as follows: denaturation at 94°C for 10 s, annealing at 61 or 62°C for 15 s and extension at 72°C for 20 s. Data were analyzed using the 2^−ΔΔCt^ method and Applied Biosystems Prism software according to the manufacturer's instructions. Whether a single temperature dissociation curve was obtained and the amplification product had the expected size were checked for each primer pair.

### Western blotting assay

The tissues of RAAs were homogenized and the total proteins were extracted by an ice-cold grinding buffer, RIPA lysis buffer (Beyotime Biotech. Co., China). Tissue lysates were boiled for 10 min with the sample buffer containing 0.062 mol/L Tris-HCl, 2% SDS, and 10% glycerol. Protein concentrations were determined using a micro BCA Protein Assay Kit (Pierce). Different samples with an equal amount of the denatured protein (20 µg/lane) were loaded, separated on 12% SDS-PAGE, and transferred to PVDF membranes. After blocking with 5% non-fat dry milk in Tris-buffered saline with 0.1% Tween-20 (TBST) for 1 h at room temperature, the membranes were incubated overnight at 4°C with a mouse monoclonal antibody against TLR2 (ab9100, Abcam, USA) at a dilution of 1:50, or a rabbit polyclonal antibody against interleukin (IL)-6 (bs-0781R, Biosynthesis, Beijing) at a dilution of 1:500, or a rabbit antibody against β-actin (#4967, Cell Signaling Technology, USA) at a dilution of 1:5000 in TBST with 5% non-fat dry milk. After 3 washes with TBST buffer (15 min each), the membranes were incubated for 1 h at room temperature with an anti-mouse or an anti-rabbit IgG secondary antibody at a dilution of 1:10000. After 3 additional washes, protein expression was detected with an enhanced chemiluminescence detection system, ECL Western Blot Detection Kit (Amersham, The Netherlands) and exposed on an X-ray Kodak film. β-actin was used as a loading control. The optical densities of TLR2, IL-6 and β-actin bands on the X-ray film were quantitatively analyzed using the Discovery Series image analysis software (Bio-Rad, USA).

### Immunohistochemistry

For immunohistochemistry analysis, tissue specimens of the RAAs were fixed in 4% formalin buffered at pH 7.0 for 24 h, and then dehydrated *via* alcohol, and embedded in paraffin. Subsequently, the paraffin-embedded sections (4-µm thick) were deparaffinized with xylene and rehydrated with gradient alcohol. The endogenous peroxidase activity was quenched for 10 min in 3% H_2_O_2_. After washing in PBS, sections were incubated overnight at 4°C with a mouse monoclonal antibody against TLR2 (1:50, ab9100, Abcam, USA). HRP-conjugated goat anti-mouse IgG was used as a secondary antibody. The horseradish-peroxidase reaction was detected by incubation with diaminobenzidine (0.05% in PBS). Sections were air dried, dehydrated through gradient alcohol and mounted for viewing.

### Statistical analysis

All statistical analyses were performed using the SPSS 17.0 software package for Windows (SPSS Inc., USA). Data were expressed as the mean±SD. For the comparison among groups, one-way ANOVA test was used for normally distributed continuous variables and the Mann-Whitney test (2 groups) and Kruskal-Wallis test (*n* groups) were applied to non-normally distributed continuous variables. In the case of categorical variables, the chi-square test or Fisher's exact test was used. Pearson's correlation was used to assess the association between TLR2 and IL-6 protein expression. A value *P* < 0.05 was considered to be statistically significant.

## RESULTS

### Patient characteristics

No significant differences existed in age and sex distribution among the three groups. The comparison of perioperative and operative characteristics is shown in ***Table 1***.

### Expression and distribution of TLR2

**Fig. 1:**
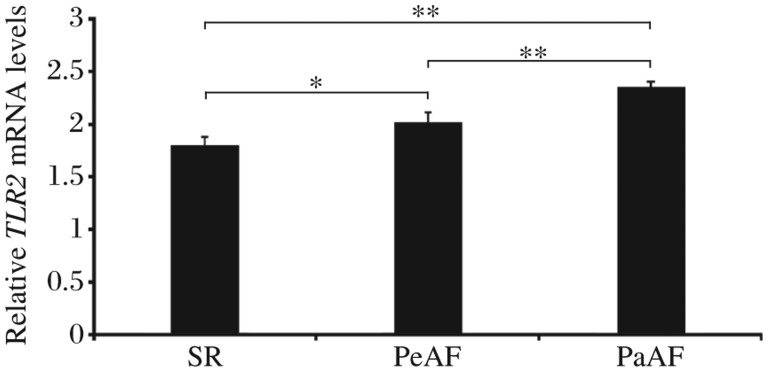
Expression of TLR2 mRNA relative to GAPDH in the right atrial appendage tissues of PaAF, PeAF and SR patients with valvular heart disease, quantified by quantitative real-time PCR. *P < 0.05, **P < 0.01. PaAF, paroxysmal atrial fibrillation; PeAF, persistent atrial fibrillation; SR, sinus rhythm.

**Fig. 2:**
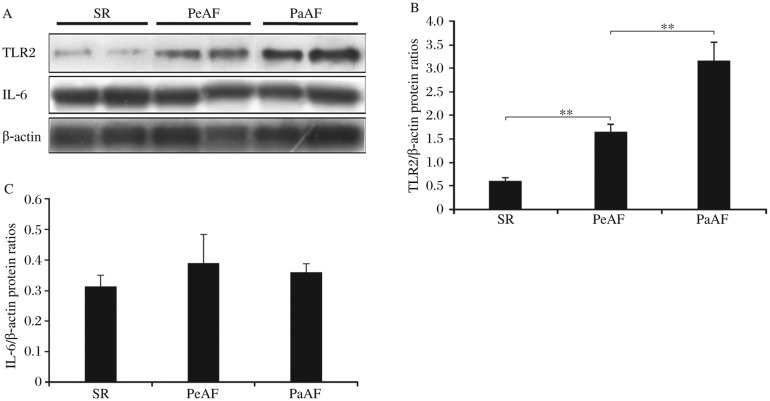
Representative Western blotting of TLR2, IL-6, and β-actin in PaAF, PeAF and SR patients with valvular heart disease. A: Western blotting results of TLR2 and IL-6 levels in different groups. Semi-quantification of TLR2 (B) and IL-6 (C) protein levels in PaAF, PeAF and SR patients with valvular heart disease determined by Western blotting against β-actin (**P < 0.01). PaAF: paroxysmal atrial fibrillation; PeAF: persistent atrial fibrillation; SR: sinus rhythm.

**Fig. 3:**
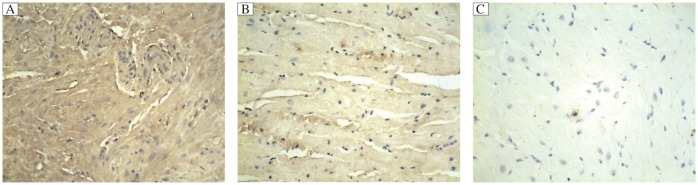
Representative photomicrographs of immunohistochemistry of TLR2 in the right atrial appendages. Gradually decreased levels of TLR2 (stained brown only in the cytoplasm) were observed in PaAF (A), PeAF (B), and SR(C) patients with valvular heart disease (original magnification, ×200).

TLR2 could be detected in the RAA tissues of AF and SR patients with valvular heart disease. The mRNA transcripts of TLR2 were elevated in patients with PaAF and PeAF, compared with those in patients with SR (***Fig. 1***). PaAF patients had a significantly higher expression of the *TLR2* gene than that in patients with PeAF. Western blotting analysis showed a significantly increasing trend of TLR2 protein levels in SR, PeAF and PaAF patients (***Fig. 2B***), which was confirmed by immunohistochemical analysis (***Fig. 3***). It also showed that the distribution pattern of TLR2 in the RAAs was different between PaAF and PeAF patients.

### Correlation between atrial TLR2 and IL-6 levels

**Fig. 4:**
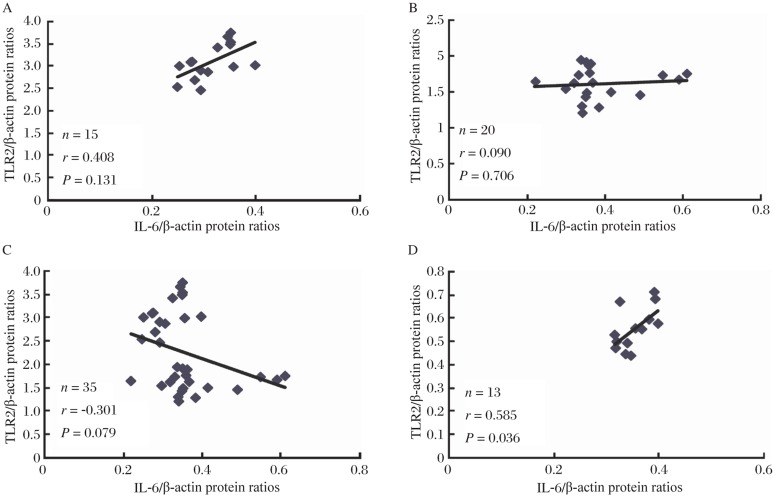
Correlation of TLR2 protein levels with IL-6 protein levels. The correlation is shown between the TLR2 value (Y-axis) and IL-6 level (X-axis) at the same time point in each valvular patient. Number of patients (n), index of correlation (r) and *P*-value (P) are indicated in the correlation charts. Dots represent individual cases. No significant correlation between TLR2 and IL-6 protein levels was identified in PeAF patients (A) or PeAF patients (B), or AF patients (C). A correlation was found between TLR2 and IL-6 in SR patients (D), *P < 0.05. PaAF: paroxysmal atrial fibrillation; PeAF: persistent atrial fibrillation; SR: sinus rhythm.

No difference of atrial IL-6 levels was observed among the three groups (***Fig. 2C***), and no correlation was found between TLR2 and IL-6 levels in PaAF patients (*r* = 0.408, *P* = 0.131, ***Fig. 4A***), PeAF patients (*r* = 0.090, *P* = 0.706, ***Fig. 4B***), and AF patients (*r* = -0.301, *P* = 0.079, ***Fig. 4C***). In contrast, a strong correlation was found between TLR2 and IL-6 in the SR group (*r* = 0.585, *P* = 0.036, ***Fig. 4D***).

## DISCUSSION

TLR2, a principal receptor for recognition of pathogen-associated molecular patterns (PAMPs), has been identified to play a crucial role in the induction and progression of chronic inflammatory disorders such as asthma[22], pulmonary inflammation and fibrosis[23], intestinal inflammation[24], dermatomyositis and polymyositis[25], and rheumatoid arthritis[26]. However, TLR2, demonstrated by emerging evidence from the past few years, appears capable of modulating inflammation and tissue damage following non-infectious insults in various tissues, such as the heart[27]–[29].

In our series, rheumatic heart disease was apparently involved in most of the cases (*n* = 41). In the remaining 7 cases with degenerative valvular disease, there were 1 with SR, 3 with PeAF, and 3 with PaAF, respectively. Indeed, it was usually possible to correlate roughly the level of TLR2 with the degree of active rheumatic inflammation. Although the presence of inflammation was associated with rheumatic activity, no significant difference was found with regard to the proportions of the etiology of valvular diseases among the three groups (*P* = 0.653). To a certain extent, the influence of rheumatic inflammation was equal. Moreover, we detected the levels of antistreptolysin O (ASO) and erythrocyte sedimentation rates (ESR) in serum at admission, which have been widely used in assessing the degree of inflammatory activity in rheumatic fever. We found that all the cases had normal plasma levels of ASO (<200 U) and ESR (<10 mm/h). It suggested a relative stabilization of rheumatic inflammatory activity in these patients in the current study. Hence, the inflammation indicated by the up-regulation of TLR2 in the atria is more likely due to AF, but not rheumatic fever.

A recent study of patients with lone AF showed that atrial biopsies revealed higher amounts of atrial fibrosis, inflammatory infiltrates, and myocyte necrosis compared to those with SR[30]. Thus, it is tempting to speculate that inflammation plays a causal role in structural remodeling, which promotes further persistence of AF. Moreover, the observations from a positive effect of corticosteroid treatment in decreasing new-onset AF in a randomized study of 216 patients undergoing valvular heart surgery[31], and a decreased incidence of AF after cardiac surgery among 241 consecutive patients without prior AF or atrial flutter[32] suggested that the interplay of inflammation may not only be a response to patients with valvular heart disease but also an integral part of AF. More recently, Hitoshi *et al*. demonstrated that TLR2 levels in the non-valvular AF group were significantly higher than those in the SR group, which implied that an infectious inflammation might participate in the pathogenesis of non-valvular AF[27]. In the present study, we also showed the novel association of AF with elevated TLR2 in the RAAs from patients with valvular heart disease. The precise mechanism is unknown, but activated TLR2 may recruit downstream IRAK4/1 via a Mal/MyD88-dependent mechanism, and ultimately lead to the activation of NF-κB and production of proinflammatory cytokines. As a supplementary, a study by Qu *et al*.[33] recently demonstrated that patients with valvular AF had higher NF-κB activity and severe lymphocyte and monocyte infiltration, which contributed to atrial structural remodeling and the incidence and maintenance of AF. This study further suggested the presence of inflammation in local atrial myocardium with elevated TLR2 level via the NF-κB pathway.

In subgroup analyses, higher TLR2 levels were found in patients with PaAF, compared with PeAF. TLR2 may be related to the “burden” or type of AF. In patients with PaAF, acute inflammatory responses and acute tissue injury may occur early in a TLR2-dependent manner. The signaling cascade mediated by TLR2 is triggered to lead to the production of inflammatory cytokines and the induction of cardiomyocyte apoptosis with a rapid up-regulation of TLR2 level in a short time. As the process extends, inflammatory states may become mild, and these paroxysms of AF may become more frequent, longer, or even persistent, potentially resulting from structural and/or electrical remodeling of the atria. TLR2 falls to a low, but plateau level, and induces very modest cardiomyocyte apoptosis in patients with PeAF. This suggests that TLR2 may be an exquisitely sensitive marker of inflammation and tissue damage, and a critical element in the transition between paroxysmal and persistent AF. Indeed, neither an inflammatory basis to valvular AF nor a causal role of TLR2 can be concluded from the present results. Nevertheless, the levels of TLR2 per se changed with the development of AF in patients with valvular disease and continued to be significantly higher in patients with PeAF compared to normal individuals. Thus, if TLR2 plays a role in AF, it may be more possible for TLR2 to promote the persistence of AF.

This study also evaluated the expression of IL-6. IL-6 is an inflammatory mediator released by immune competent cells, which can initiate specific and nonspecific inflammatory reactions, and is described to exert both proinflammatory and anti-inflammatory effect, depending on cell type and the specific physiologic and pathologic state[34]. Qu *et al*.[33] has demonstrated that patients with valvular AF had higher concentration of IL-6 than patients with SR. However, IL-6 levels were similar among the three groups in our study, which implied that IL-6 may be less specific compared with TLR2 for inflammation in valvular AF. Another explanation is that TLR2 elevation might be independent of other risk factors. Though there is limited evidence about the role and functional pathway of TLR2 in valvular AF, TLR2 elevation should at least be a predictive signal of AF pathogenesis and development. Thus, it is possible that markedly increased TLR2 levels may have a direct role in mediation of locally inflammatory effects on AF, whereas IL-6 level is mainly responsible for a state of systemic inflammation.

In conclusion, TLR2, independent of IL-6, was up-regulated in AF (especially PaAF) patients with valvular heart disease, which further implicates inflammation in the pathogenesis and development of AF. However, firstly, the small sample size limited the statistical power of our study and large well-designed studies are warranted to confirm our findings. Secondly, because we only used the statistical method to infer the haplotypes, future functional studies are warranted to investigate the precise roles of TLR2 in AF, elucidate the precise molecular mechanisms and exploit its potential therapeutic purpose.
